# Licensing Medical Practitioners

**Published:** 1888-05

**Authors:** Frank Billings

**Affiliations:** 235 State Street


### DOMESTIC CORRESPONDENCE.

To the Editor: Recent issues of many medical journals contain complaints from medical editors and contributors of the over-crowded condition of the profession, and of the continued annual addition of numerous graduates from the colleges increasing the plethora. "Diploma Mills," "Diploma-mill Professors," and other uncomplimentary terms are indulged in to designate, seemingly, all medical colleges and all medical teachers.

It is not the purpose of this article to combat these complaints, for it is quite universally conceded that the medical profession is over-crowded; that the mode of teaching in many colleges is lax, and the time of study too short in nearly all medical colleges; and that too many are annually graduated and *licensed by the diplomas they hold* to at once practice medicine. It is true, too, that many students are received in some colleges who are, by reason of a lack of even a reasonable knowledge of English, quite unprepared to study medicine. But will this condition of affairs be removed, or even improved, by the methods of attack mentioned above?

It seems improbable as long as a diploma alone is accepted as entitling the holder of it to practice, or it is accepted by the licensing board of a State as sufficient evidence of qualification. Minnesota has fairly indicated what should be done. The licensing power of that State is a State Board of Medical Examiners, the members of which are not connected in any way with a medical college.

This board requires of all who desire to begin the practice of medicine in the State, a satisfactory examination in medicine and surgery. Only those are eligible to such an examination who have studied medicine for three years, during which time they must have attended *three annual* courses of lectures of *at least* six months each. A similar law regulates the practice of medicine in California. A few other States have laws which in some measure approach that of Minnesota, but which are still faulty. Our own State Board of Health has done much to elevate the standard of medical education in Illinois; but we need an examining board similar to that of Minnesota. No so-called school of medicine should be recognized. No one connected in any way with a medical college should be eligible to membership on the board. The requirements of eligibility to these examinations should be not less than in Minnesota —that is, not less than three years of study —which should include attendance upon three annual courses of lectures of not less than six months each, in a college which requires and enforces a good English education and accompanying mental discipline, preliminary to the study of medicine. With such requirements from every man the prospect of elevating the standard of medical education and requirement will be materially increased.

The difficulties in the way of securing a diploma, and with it a license to practice, will be so increased that fewer men will attempt it—the ones least prepared to study will drop out. A diminution in the number of students will necessarily close such colleges as depend for support upon those who study medicine because a diploma is easily secured. There are colleges which will support, and aid in securing, this needed reform. Let the general profession take the initative step at once in local and State societies. If a draft of a bill be agreed upon and the influence of the physicians in the State be brought to bear on their representatives in the legislature, desired legislation may be secured.

We have indicated the outlines of the only method of reform in medical education. As occasion requires we shall discuss the subject more minutely. We ask those members of the profession, and especially our medical contemporaries, who have spent their efforts in vainly decrying medical colleges and medical college professors, to join with us in securing needed reform. And one of the most effective means to that end would make all medical colleges simply teaching bodies, granting no diplomas which should imply a right to practice medicine.

235 State Street. Frank Billings.

